# *In Utero* Fine Particle Air Pollution and Placental Expression of Genes in the Brain-Derived Neurotrophic Factor Signaling Pathway: An ENVIR*ON*AGE Birth Cohort Study

**DOI:** 10.1289/ehp.1408549

**Published:** 2015-03-27

**Authors:** Nelly D. Saenen, Michelle Plusquin, Esmée Bijnens, Bram G. Janssen, Wilfried Gyselaers, Bianca Cox, Frans Fierens, Geert Molenberghs, Joris Penders, Karen Vrijens, Patrick De Boever, Tim S. Nawrot

**Affiliations:** 1 Centre for Environmental Sciences, Hasselt University, Diepenbeek, Limburg, Belgium; 2 Department of Physiology, Hasselt University, Diepenbeek, Limburg, Belgium; 3 Department of Obstetrics, East-Limburg Hospital, Genk, Limburg, Belgium; 4 Belgian Interregional Environment Agency, Brussels, Brussels Capital Region, Belgium; 5 I-BioStat, Hasselt University, Diepenbeek, Limburg, Belgium; 6 I-Biostat, Leuven University (KU Leuven), Leuven, Flemish Brabant, Belgium; 7 Laboratory of Clinical Biology, East-Limburg Hospital, Genk, Limburg, Belgium; 8 Unit Environmental Risk and Health, Flemish Institute for Technological Research, Mol, Antwerp, Belgium; 9 Department of Public Health and Primary Care, Leuven University (KU Leuven), Leuven, Flemish Brabant, Belgium

## Abstract

**Background:**

Developmental processes in the placenta and the fetal brain are shaped by the same biological signals. Recent evidence suggests that adaptive responses of the placenta to the maternal environment may influence central nervous system development.

**Objectives:**

We studied the association between *in utero* exposure to fine particle air pollution with a diameter ≤ 2.5 μm (PM_2.5_) and placental expression of genes implicated in neural development.

**Methods:**

Expression of 10 target genes in the brain-derived neurotrophic factor (*BDNF*) signaling pathway were quantified in placental tissue of 90 mother–infant pairs from the ENVIR*ON*AGE birth cohort using quantitative real-time polymerase chain reaction. Trimester-specific PM_2.5_ exposure levels were estimated for each mother’s home address using a spatiotemporal model. Mixed-effects models were used to evaluate the association between the target genes and PM_2.5_ exposure measured in different time windows of pregnancy.

**Results:**

A 5-μg/m^3^ increase in residential PM_2.5_ exposure during the first trimester of pregnancy was associated with a 15.9% decrease [95% confidence interval (CI): –28.7, –3.2%, *p* = 0.015] in expression of placental *BDNF* at birth. The corresponding estimate for synapsin 1 (*SYN1*) was a 24.3% decrease (95% CI: –42.8, –5.8%, *p* = 0.011).

**Conclusions:**

Placental expression of *BDNF* and *SYN1*, two genes implicated in normal neurodevelopmental trajectories, decreased with increasing *in utero* exposure to PM_2.5_. Future studies are needed to confirm our findings and evaluate the potential relevance of associations between PM_2.5_ and placental expression of *BDNF* and *SYN1* on neurodevelopment. We provide the first molecular epidemiological evidence concerning associations between *in utero* fine particle air pollution exposure and the expression of genes that may influence neurodevelopmental processes.

**Citation:**

Saenen ND, Plusquin M, Bijnens E, Janssen BG, Gyselaers W, Cox B, Fierens F, Molenberghs G, Penders J, Vrijens K, De Boever P, Nawrot TS. 2015. *In utero* fine particle air pollution and placental expression of genes in the brain-derived neurotrophic factor signaling pathway: an ENVIR*ON*AGE Birth Cohort Study. Environ Health Perspect 123:834–840; http://dx.doi.org/10.1289/ehp.1408549

## Introduction

Ambient air pollution is a global public health threat (Nawrot et al. 2011). Recent evidence suggests that *in utero* exposure to particulate matter with a diameter ≤ 2.5 μm (PM_2.5_) affects placental functional morphology in mice (Veras et al. 2008), as well as normal fetal development in humans because of suboptimal intrauterine environment (Ballester et al. 2010).

David Barker introduced the concept that early-life stress contributes to later illness (Barker 1990). Perturbations in the maternal environment can be transmitted to the fetus by changes in placental function. This might affect fetal programming and thereby increase the risk of cardiovascular disease later in life (Jansson and Powell 2007). Furthermore, recent findings show increasing support for effects of environmental exposures on diseases of the central nervous system (Block and Calderón-Garcidueñas 2009).

The neurodevelopmental trajectories of the fetal brain are vulnerable processes that may be disturbed by toxic insults and potentially by *in utero* exposures to air pollution. Experimental evidence obtained in mice shows that prenatal diesel exposure affects behavior (Bolton et al. 2013), neurotransmitter levels, and spontaneous locomotor activity (Suzuki et al. 2010). A prospective cohort study reported that children with higher prenatal exposure to ambient polycyclic aromatic hydrocarbons had a lower IQ at 5 years of age (Edwards et al. 2010). Suglia et al. (2008) reported that exposure to black carbon was associated with reduced cognitive function scores in 8- to 11-year-old children. Although both experimental and epidemiological evidence suggests that exposure to fine particle air pollution affects the brain of offspring in the developmental period, potential mechanisms that may underlie such early-life changes have not been characterized.

**Figure 1:**
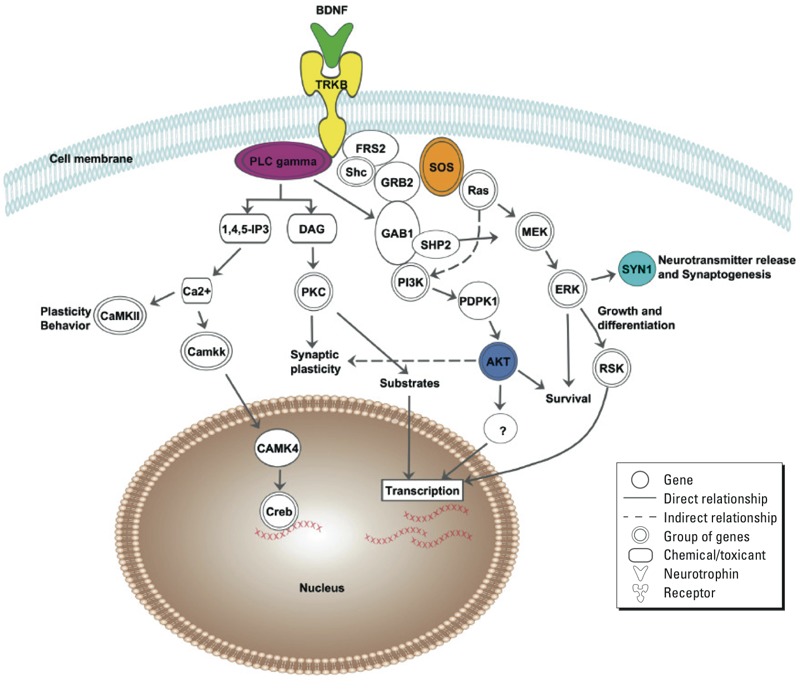
Overview of the genes within the *BDNF* signaling pathway [adapted with permission from Macmillan Publishers Ltd. (Minichiello 2009)]. The binding of BDNF to its receptor TRKB initiates three main signaling cascades: PLC gamma cascade (*PLCG1* and *PLCG2*), AKT cascade (*AKT1*, *AKT2*, and *AKT3*) and SOS cascade (*SOS1*, *SOS2*, and *SYN1*). These cascades are involved in neuronal survival, growth, differentiation, and synaptic plasticity. The highlighted genes were explored in this study.

Two recent studies (Bonnin et al. 2011; Broad and Keverne 2011) suggest that the placenta, aside from transport of maternal nutrients, growth factors, and hormones, also plays an important role in central nervous development through adaptive responses to the maternal environment. Neurotrophins are implicated in a host of brain cellular functions. Multiple experimental studies have shown that brain-derived neurotrophic factor (BDNF) plays a role in development and function of the nervous system, which includes also the thyroid hormone–brain development axis (Gilbert and Lasley 2013). Moreover, it has been suggested that maternal BDNF is able to reach the fetal brain through the utero-placental barrier in mice and may therefore contribute to the development of the fetal central nervous system (Kodomari et al. 2009). Recently, cord blood BDNF levels were positively associated with scores on Gesell Development Schedules at 2 years of age among children enrolled before and after the closure of a coal-fired power plant in Tongliang County, China (Tang et al. 2014). In this context, we studied placental expression of genes in the *BDNF* signaling pathway (Figure 1) (Minichiello 2009). *BDNF* is expressed in the central and peripheral nervous system and in tissues/organs where it regulates morphogenesis, proliferation, apoptosis, and developmental processes (Sariola 2001). An *in vitro* study showed that BDNF and its specific receptor, tyrosine kinase (TRKB), are also involved in embryo implantation, subsequent placental development, and fetal growth by stimulating trophoblast cell growth and survival. Moreover, BDNF promotes neuronal maturation and differentiation of the developing nervous system (Tometten et al. 2005) and participates in synaptogenesis (Cohen-Cory et al. 2010). For example, BDNF modulations of neurotransmitter release in mice can alter the activity of synapsin 1 (SYN1). The latter protein promotes axonal growth and neuroplasticity, helps to maintain synaptic contacts, and influences synaptic vesicle exocytosis via a mitogen-activated protein kinase (MAPK)–dependent phosphorylation (Jovanovic et al. 2000).

Environmental factors may modulate placental gene expression in a way that the fetus’ normal neurodevelopmental trajectory is affected. In the present study, we investigated whether *in utero* exposure to PM_2.5_ during different periods of prenatal life is associated with placental expression of neurodevelopmental genes in the *BDNF* signaling pathway at birth.

## Methods

*Study population and measurements.* The ongoing ENVIR*ON*AGE birth cohort enrolls mothers giving birth in the East-Limburg Hospital (ZOL; Genk, Belgium). The hospital has a catchment area of 2,422 km^2^ and includes rural, suburban, and urban municipalities with population densities ranging from 82 to 743 inhabitants/km^2^. From February 2010 through March 2012, we recruited mother–newborn pairs (only singletons) born between Friday 1200 hours and Monday 0700 hours. Enrollment was spread equally over all seasons of the year. The participation rate of eligible mothers (able to fill out a Dutch language questionnaire) was 56% (*n* = 320). Most common reasons for nonparticipation of eligible mothers were recorded during the first month of the campaign: *a*) failure to ask for participation, *b*) communication problems, and *c*) complications during labor. In the present study, exclusion criteria based on exposure to active or passive tobacco smoking reduced the study population to 247 participants. From this smoke-free group, a random selection of 90 mother–newborn pairs was used for gene expression analysis. A comparison of our subsample with the full cohort and with the Flemish birth register (Cox et al. 2013) did not show significant differences in maternal age, pregestational body mass index (BMI), parity, ethnicity, birth weight, and birth length. The study was approved by the Ethics Committee of Hasselt University and East-Limburg Hospital. Written informed consent was obtained from all participating mothers when they arrived at the hospital for delivery. Study questionnaires providing detailed information on place of residence, age, pregestational BMI, net weight gain during pregnancy, maternal education, occupation, smoking status, alcohol consumption, use of medication, parity, and neonates’ ethnicity were completed in the postnatal ward after delivery. Perinatal parameters such as neonates’ sex, birth date, birth weight and length, gestational age, Apgar score, and ultrasonographic data were also collected after birth. Gestational age was estimated based on ultrasound data. Insulin levels were measured in cord blood using the E-modular 170 (Roche Diagnostics, Vilvoorde, Belgium).

*Placental tissue.* Placentas were collected within 10 min after birth. Biopsies were taken at four standardized sites across the middle region of the fetal side of the placenta, approximately 4 cm away from the umbilical cord. Two biopsies were used in our analysis. The first biopsy was taken to the right of the main artery, the second in the third quadrant of the placenta. We sampled 1.0–1.5 cm below the chorio-amniotic membrane at a fixed location. Tissue samples were transferred to RNALater (Qiagen, KJ Venlo, the Netherlands) and incubated at 4°C for 24 hr. Samples were archived at –20°C.

*RNA extraction.* Samples were thawed and RNA was extracted from 20 to 25 mg placental tissue using the miRNeasy Mini Kit (Qiagen). Genomic DNA contamination was minimized with the Turbo DNA free kit (Ambion, Life Technologies, Foster City, CA, USA). The concentration of total RNA was measured with Nanodrop spectrophotometer (ND-1000; Isogen Life Science, De Meern, the Netherlands). The average yield ± SD of total RNA per placenta biopsy was 8.8 ± 3.5 μg with A_260/280_ ratio of 1.98 ± 0.05 and A_260/230_ ratio of 1.75 ± 0.22. Extracted RNA was stored at –80°C until further use.

*Gene expression analysis.* Expression of candidate genes (*n* = 10) within the *BDNF* signaling pathway was studied (see Supplemental Material, Table S1). Candidate genes were selected based on literature with regard to neurodevelopment (Figure 1). A maximum amount of 3 μg of total RNA was reverse transcribed into cDNA by means of the GoScript Reverse Transcription System (Promega, Madison, WI, USA) using a Veriti 96-well Thermal cycler (TC-5000; Techne, Burlington, NJ, USA). cDNA was stored at –20°C until further measurements. A quantitative real-time polymerase chain reaction (qPCR) was set up by adding 2 μL of a 10-ng/μL dilution of cDNA together with TaqMan Fast Advanced Master Mix (Life Technologies) and PrimeTime^TM^ assay (Integrated DNA Technologies, Coralville, IA, USA) in a final reaction volume of 10 μL. Standard cycling conditions were used to analyze samples in a 7900HT Fast Real-Time PCR system (Life Technologies). Cq values were collected with SDS2.3 software. MIQE (minimum information for publication of quantitative real-time PCR experiments) guidelines were taken into account (Bustin et al. 2009). Amplification efficiencies were between 90 and 110% for all assays (see Supplemental Material, Table S1), and amplification specificity was confirmed by gel electrophoresis (data not shown). Raw data were processed to normalized relative gene expression values with qBase plus software (Biogazelle, Zwijnaarde, Belgium) using *IPO8*, *POLR2A*, *UBC*, and *GAPDH* as reference genes for data normalization (see Supplemental Material, Table S1). Technical replicates were included when the difference in Cq value was < 0.75. The correlation coefficient of gene expression between the two biopsies varied between 0.29 for *SYN1* and 0.85 for *AKT1* (data not shown). Between-placenta variability was higher than within-placenta variability for all genes, except for *SYN1*, *AKT2,* and *PLCG2* (see Supplemental Material, Table S2).

*Exposure estimates.* Regional background levels of PM_2.5_ were interpolated for each mother’s residential address using a spatiotemporal interpolation method (kriging) that uses land cover data obtained from satellite images (Corine land cover data set) in combination with monitoring stations (*n* = 34) (Janssen et al. 2008; Maiheu et al. 2013). This model provides interpolated PM_2.5_ values from the Belgian telemetric air quality networks in 4 × 4 km grids. Based on 34 different locations, validation statistics of the interpolation tool gave a temporal explained variance of > 0.8 for hourly PM_2.5_ averages as well as for annual mean PM_2.5_. Additionally, nitrogen dioxide (NO_2_) exposures were interpolated using the same methods as PM_2.5_ exposure. To explore potentially critical exposure windows during pregnancy, the daily interpolated PM_2.5_ concentrations (micrograms per cubic meter) were averaged for various periods during pregnancy for which the date of conception was estimated based on ultrasound data (Janssen et al. 2013)—that is, the three trimesters (1–13 weeks, 14–26 weeks, and 27 weeks to delivery) and the early pregnancy stages: preimplantation (1–5 days after estimated conception date), implantation (6–12 days), implantation range (6–21 days, imbedding of blastocyst in endometrium), postimplantation (22–28 days), and first month (1–30 days). Mean daily temperatures and relative humidity for the study region were provided by the Royal Meteorological Institute (Brussels, Belgium).

*Statistical analysis.* Statistical analysis was carried out using SAS software (version 9.3; SAS Institute Inc., Cary, NC, USA). Continuous data were presented as mean ± SD and categorical data as frequencies and percentages.

In a first (single-gene) analysis, we examined the association between gene expression of two placenta biopsies and PM_2.5_ exposure. The correlation between the two biopsies from a single placenta was accounted for by using mixed-effects models (Verbeke and Molenberghs 2000). Models were adjusted for linear terms for maternal age, gestational age, cord blood insulin, delivery date, and NO_2_ exposure and indicator variables for newborn’s sex, maternal education (low, middle, high), placental biopsy site, and season at birth (winter, spring, summer, and autumn). Because both air pollution (Park and Wang 2014) and BDNF (Fujinami et al. 2008) are related to glucose metabolism, cord blood insulin was added to the models. For each exposure window, estimates are calculated for a 5-μg/m^3^ increment in PM_2.5_, and results are presented as a percent change in gene expression relative to the mean gene expression.

In a second (multiple-gene) analysis, we explored the three different signaling cascades of the *BDNF* pathway. Gene expression values of genes belonging to the same cascade were treated as a single outcome and were entered into a mixed model. Within the *AKT* cascade, the response variable consisted of eight correlated gene expression values for each placenta—that is, two biopsies per placenta and four target genes (*BDNF*, *AKT1*, *AKT2,* and *AKT3*) measured in each biopsy. Similarly, the response variable consisted of eight gene expression values per placenta within the *SOS* cascade (*BDNF*, *SOS1*, *SOS2,* and *SYN1*) and six gene expression values per placenta within the *PLCG* cascade (*BDNF*, *PLCG1,* and *PLCG2*). *TRKB* was not significantly correlated with the other transcript levels within these cascades and therefore was excluded from this analysis (see Supplemental Material, Table S3). The mixed model adjusts for the correlation between the biopsies and for the correlation between the genes from a single placenta, whereas differences between genes are accounted for by entering them as a fixed effect into the model. Models were adjusted for the same confounders or covariates as in the single-gene analyses. The assumption that the effect of the exposure was the same across all target genes within a cascade was assessed by including interaction terms between gene and exposure. Results are presented as a difference in gene expression for a 5-μg/m^3^ increment in PM_2.5_ for each exposure window.

## Results

*Study population characteristics and exposure levels.* Demographic characteristics of the 90 mother–infant pairs are presented in Table 1. Maternal age was on average ± SD 29.5 ± 4.6 years. Pregestational BMI averaged 24.1 ± 4.4 kg/m^2^ with a mean net weight gain of 15.5 ± 7.2 kg during pregnancy. Fifty-eight (64.4%) of the mothers obtained a higher education degree. The total newborn population, comprising 47 boys (52.2%), had a mean gestational age of 39.1 weeks (range, 35–42); 92.2% were term-born infants and included a vast majority of primiparous (55.6%, *n* = 50) or secundiparous (32.2%, *n* = 29) newborns. Birth weight and length were 3,450 ± 436 g and 50.5 ± 1.9 cm, respectively.

**Table 1:** Characteristics of mother–newborn pairs (*n* = 90).

Characteristic	Mean ± SD or *n* (%)
Maternal	
Age (years)	29.5 ± 4.6
Pregestational BMI (kg/m^2^)	24.1 ± 4.4
Net weight gain (kg)	15.5 ± 7.2
Mother’s education^*a*^
Low	11 (12.2)
Middle	21 (23.3)
High	58 (64.4)
Acetaminophen during pregnancy^*b*^
No	45 (54.2)
Alcohol consumption during pregnancy^*c*^	
No	79 (89.8)
Parity
1	50 (55.6)
2	29 (32.2)
≥ 3	11 (12.2)
Newborn
Sex
Male	47 (52.2)
Ethnicity^*d*^
European	74 (83.2)
Gestational age (weeks)	39.1 ± 1.3
Born at term (> 37 weeks)	83 (92.2)
Season at birth
Spring	22 (24.4)
Summer	19 (21.1)
Autumn	14 (15.6)
Winter	35 (38.9)
Apgar score after 5 min
6	1 (1.1)
7	0 (0)
8	6 (6.7)
9	25 (27.8)
10	58 (64.4)
Birth weight (g)	3,450 ± 436
Birth length (cm)	50.5 ± 1.9
Cord blood insulin (mU/L)	7.3 ± 7.3
^***a***^Mother’s education: low (no high school diploma), middle (high school diploma), high (college or university diploma). ^***b***^Data available for 83 subjects. ^***c***^Data available for 87 subjects. ^***d***^Data available for 89 subjects.	

Mean outdoor PM_2.5_ for the different time windows of pregnancy are reported in Table 2 and the values for NO_2_ are presented in Supplemental Material, Table S4.

**Table 2:** PM_2.5_ (μg/m^3^) exposure characteristics (*n* = 90).

Time windows	Mean ± SD	25th percentile	75th percentile
Preimplantation (1–5 days)	17.4 ± 10.5	10.7	20.6
Implantation (6–12 days)	17.0 ± 9.5	10.8	20.1
Implantation range^*a*^ (6–21 days)	16.5 ± 7.5	11.3	18.9
Postimplantation (22–28 days)	15.0 ± 8.4	9.6	17.2
First month (1–30 days)	15.8 ± 6.6	11.6	17.9
Trimester 1 (1–13 weeks)	15.4 ± 5.4	11.7	18.0
Trimester 2 (14–26 weeks)	17.6 ± 7.0	12.0	22.8
Trimester 3 (27 weeks–delivery)	18.7 ± 6.0	14.9	23.0
^***a***^Data available for 79 subjects.			

**Figure 2:**
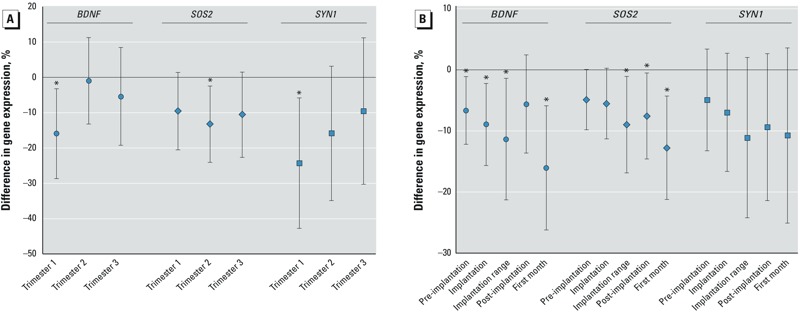
Difference in *BDNF*,* SOS2*, and *SYN1* placental gene expression in association with *in utero* exposure to fine particle air pollution (PM_2.5_) during various time windows (single-gene models; *n* = 90). The effect estimate is the percent difference (95% CI) relative to mean gene expression for a 5-μg/m^3^ increase of PM_2.5_ exposure (μg/m^3^). Time window–specific PM_2.5_ exposures (μg/m^3^) were calculated by averaging the daily interpolated PM_2.5_ concentrations for various periods during pregnancy: each of the three trimesters (*A*) and the early pregnancy stages (*B*). Estimates were adjusted for newborn’s sex, maternal age, maternal education, gestational age, cord blood insulin, placental biopsy site, delivery date, season at birth, and NO_2_ exposure. *p < 0.05.

*Gene expression of the* BDNF *signaling pathway in association with PM_2.5_ exposure: single-gene models.* Placental *BDNF* gene expression was inversely associated with PM_2.5_ exposures during the first trimester of pregnancy: placental *BDNF* expression decreased by 15.9% [95% confidence interval (CI): –28.7, –3.2%, *p* = 0.015] for a 5-μg/m^3^ increment in PM_2.5_ (Figure 2A). This association was adjusted for newborn’s sex, maternal age, maternal education, gestational age, cord blood insulin, placental biopsy site, delivery date, season at birth, and NO_2_ exposure. We observed no significant association between *BDNF* expression and PM_2.5_ exposure in the second (*p* = 0.88) and third trimesters (*p* = 0.44). In a second stage, we examined shorter time windows to target more specifically the critical stages of placental and fetal development, and estimated a significant negative association of placental *BDNF* gene expression with PM_2.5_ exposure during the first month of pregnancy and during early implantation stages (Figure 2B). For the postimplantation window, the negative association weakens with loss of statistical significance. Significant inverse associations were found between *SYN1* and PM_2.5_ during trimester 1 and between *SOS2* and PM_2.5_ during trimester 2 [–24.3% (95% CI: –42.8, –5.8%, *p* = 0.011) and –13.3% (95% CI: –24.1, –2.4%, *p* = 0.017) for a 5-μg/m^3^ increment in PM_2.5_ respectively] (Figure 2A). Within the shorter time windows, significant associations were observed between *SOS2* gene expression at birth and PM_2.5_ exposure during several implantation stages and the first month of pregnancy (Figure 2B). No significant associations were found between PM_2.5_ exposure and other selected genes within the *BDNF* pathway (see Supplemental Material, Figure S1).

BDNF *signaling cascades in association with PM_2.5_ exposure: multiple-gene models.* To test the assumption that the effect of the exposure was the same across all target genes within a cascade, we used an interaction term between the exposure and the variable identifying the gene. Because interaction terms were not significant, they were excluded from final models. We found for the *PLCG* cascade (*BDNF, PLCG1,* and *PLCG2*) that PM_2.5_ exposure during the first month (*p* = 0.001) and the first trimester (*p* = 0.009) of pregnancy was associated with significantly lower levels of placental gene expression at birth (Table 3). We also observed significant changes in gene expression in association with PM_2.5_ exposure during the first month (*p* = 0.00002) and first trimester of pregnancy (*p* = 0.0001) for the *SOS* cascade (*BDNF*, *SOS1*, *SOS2,* and *SYN1*), whereas for the *AKT* cascade (*BDNF*, *AKT1*, *AKT2,* and *AKT3*) associations between gene expression and PM_2.5_ exposure were not significant (Table 3).

**Table 3:** Associations between cascade-specific placental gene expression and PM_2.5_ exposure during pregnancy (multiple-gene models) (*n* = 90).

Time windows	*AKT* cascade *BDNF, AKT1, AKT2, AKT3*		*SOS* cascade *BDNF, SOS1, SOS2, SYN1*		*PLCG* cascade *BDNF, PLCG1, PLCG2*	
	β (95% CI)	*p*-Value	β (95% CI)	*p*-Value	β (95% CI)	*p*-Value
First month of pregnancy	–0.03 (–0.07, 0.0009)	0.06	–0.1 (–0.2, –0.07)	0.00002	–0.08 (–0.1, –0.03)	0.001
Trimester 1	–0.03 (–0.08, 0.02)	0.2	–0.15 (–0.2, –0.08)	0.0001	–0.1 (–0.2, –0.03)	0.009
Trimester 2	0.02 (–0.03, 0.07)	0.4	–0.05 (–0.2, 0.04)	0.3	0.02 (–0.09, 0.1)	0.7
Trimester 3	–0.03 (–0.09, 0.02)	0.2	–0.09 (–0.2, 0.02)	0.1	–0.09 (–0.2, 0.01)	0.08
In three separate models, estimates (95% CI) express the multivariable adjusted change in gene expression for a 5-μg/m^3^ increment in PM_2.5_. Estimates were adjusted for newborn’s sex, maternal age, maternal education, gestational age, cord blood insulin, placental biopsy site, delivery date, season at birth, and NO_2_ exposure. The models account for nonindependence of placenta biopsies and genes within each cascade.						

## Discussion

Both animal and epidemiologic studies indicate that nutrition and environmental stimuli influence *in utero* developmental pathways and may even induce permanent changes in metabolism and chronic disease susceptibility (McMillen and Robinson 2005). Transcriptional changes during the perinatal period are associated with morphological and functional development of the brain (Muotri and Gage 2006). In this regard, recent studies suggest that aside from its traditional role in maternal–fetal exchange of nutrients, the placenta plays a role in neurodevelopmental processes through adaptive responses to the maternal environment (Zeltser and Leibel 2011). A recent study provided evidence of significant measureable benefits of children’s neurocognitive development and cord blood BDNF based on a comparison of two birth cohorts in Tongliang, China, with measurements before and after closure of the local power plant. The investigators found that prenatal PAH exposure was negatively associated with BDNF levels in cord blood, and that BDNF levels were associated with poorer developmental scores in children (Tang et al. 2014). Here, we demonstrated that placental *BDNF* and *SYN1* gene expression levels at birth were inversely associated with PM_2.5_ exposure levels in the first trimester of pregnancy. We surmise that an altered expression of these genetic targets could be part of a molecular mechanism through which fine particle air pollution exposure might affect placental processes.

The concept of the placental role in brain development is relatively new and in line with the groundbreaking observations of fetal programming and disease susceptibility later in life (Barker 1990). Critical developmental processes in the placenta and fetal brain are shaped by the same biological signals (Zeltser and Leibel 2011). In mice, Broad and Keverne (2011) observed a strong co-expression of imprinted genes in the hypothalamus and placenta at mid-gestation (embryonic day 11–13), an important period of neuronal proliferation and differentiation. Experimental evidence showed that both BDNF and SYN1 are involved in critical developmental processes of the nervous system, including proliferation, migration, differentiation, and synaptogenesis (Bernd 2008; Fornasiero et al. 2010). In mice, *Bdnf* signaling plays important paracrine roles during blastocyst outgrowth (Kawamura et al. 2007). It might promote the development of preimplantation embryos by suppressing apoptosis and stimulating trophoblast cell growth and survival (Kawamura et al. 2009). Furthermore, *Bdnf* appears to play an important role in ventricular progenitor cell migration in the developing mouse cerebral cortex (Ohmiya et al. 2002). In mice, the BDNF protein contributes to regulation of spontaneous correlated activity at early developmental stages by increasing synaptogenesis and expression of the K^+^/Cl^–^ co-transporter KCC2 (Aguado et al. 2003). In line with these experimental observations, we found that exposure to fine particle air pollution from the estimated day of conception up to embryo implantation was negatively associated with placental *BDNF* expression at birth. In the same cohort, total DNA hypomethylation was associated with specific PM_2.5_ exposure windows around implantation (Janssen et al. 2013). This type of epigenetic modification could be a biologically plausible link between *in utero* exposures and altered gene expression at birth.

Multiple signaling cascades (Figure 1) implicated in several neurological processes are initiated once BDNF binds to its receptor (Bhatia et al. 2011). In our study, we observed no significant correlation between the expression of *TRKB* and the expression of other selected genes in the *BDNF* signaling pathway. However, studies in mice showed that *Trkb* mRNA levels are already high during the prenatal period and that expression does not significantly fluctuate throughout development (Ivanova and Beyer 2001). The PLCG cascade, underlying BDNF, has been linked to synaptic plasticity (Li et al. 2005). Furthermore, mutations in the PLCG docking site altered hippocampal plasticity in mice by which learning was affected (Gruart et al. 2007). In the present study, we found an inverse association between gene expression within the *PLCG* cascade and PM_2.5_ exposure during the first month and during the first trimester of pregnancy. We hypothesize that differences in gene expression of the *BDNF* pathway might alter signaling and thereby neurodevelopmental processes. We also observed differences in gene expression within the *SOS* cascade in association with PM_2.5_ exposure during the first month and first trimester of pregnancy. In general, in response to upstream stimuli the SOS proteins function as enzymatic factors interacting with RAS proteins to promote guanine nucleotide exchange (GDP/GTP) followed by the formation of the active RAS–GTP complex (Rojas et al. 2011). In humans, the SOS family contains two different genes (*SOS1* and *SOS2*), located on different chromosomes. Although these genes are highly similar in structure and sequence, a study in mice demonstrated that the lack of SOS1 protein leads to embryonic death, whereas lack of SOS2 did not alter fetal growth and development (Esteban et al. 2000). Via the SOS cascade, BDNF increases Ras–MAPK–dependent phosphorylation of SYN1 (Jovanovic et al. 2000), which promotes axonal growth and neuroplasticity. In our study, placental *SYN1* gene expression was decreased with maternal exposure to fine particle air pollution during the first trimester of pregnancy. During development, the expression of *SYN1* correlates temporally and topographically with synaptogenic differentiation (Melloni et al. 1994). Animal studies revealed that during the development of the hippocampus the temporal onset and the peak expression of *Syn1* coincides with neuronal and synaptogenic differentiation of granule cell neurons (Melloni and DeGennaro 1994).

Biological mechanisms through which PM might affect the placenta and subsequent development of the fetus are uncertain. The formation of inflammatory and oxidative stressors is thought to be of importance (Risom et al. 2005). Inflammation might contribute to inadequate placental perfusion affecting nutritional processes or oxygenation of maternal blood. In addition, activation of inflammatory cells, which are capable of forming reactive oxygen species (ROS), increases oxidative stress–induced DNA damage, which appears to be a particularly important mechanism of action of PM (Risom et al. 2005). This suggests that, depending on the chemicals present on the surface of PM, two different pathways might be considered to affect the transcriptional release and operation of genes: *a*) indirectly via systemic consequences of induced inflammatory conditions both in mother’s lungs as well as in placental tissue, or *b*) via translocation of inhaled fine particles from the lung into the blood stream leading to oxidative stress in blood cells and potentially in placental tissue. In an *ex vivo* human placental perfusion model, Wick et al. (2010) showed that particles up to 240 nm in diameter can cross the placental barrier.

A problem common in molecular epidemiology studies is the need to adjust for the multiple comparisons in the analyses, which may be a first limitation of our study. We have done multiple statistical analysis to identify associations of different genes in the *BDNF* signaling pathway and the different exposure windows. However, overall our analysis finds consistent results with the strongest effect for *BDNF*. A second limitation is that our small sample has an overrepresentation of higher-educated women, probably because we excluded mothers exposed to tobacco smoke (both active and passive). Therefore the generalizability of our findings may be limited. However, this methodological consideration was deliberately applied to decrease the risk of potential residual confounding in smaller samples. A third limitation of the present study is the complexity of the placenta tissue. Because the placenta is composed of different cells including syncytiotrophoblasts, mesenchymal cells, and fibroblasts as well as maternal blood and cord blood, the within-placenta variability is high (Adibi et al. 2010). Sample composition of each biopsy can differ considerably, and this can influence gene expression patterns. To minimize biopsy to biopsy variation, we standardized our biopsy method by taking two fetal-side biopsies. Observational population studies allow us to characterize only associations between exposure and biomarkers of effect using noninvasive methods, and this may be a fourth limitation of our study. It might be that our observations are functionally not related to *in utero* neurodevelopment, but reflect placental function and development in general. However, recent experimental evidence suggests that the placenta might be a useful surrogate tissue to explore fetal brain development (Zeltser and Leibel 2011).

## Conclusions

In our study population, estimated *in utero* PM_2.5_ exposure during the first trimester of pregnancy was negatively associated with the placental transcription of *BDNF* and *SYN1*, two genes implicated in neural development. Average estimated PM_2.5_ exposures in our study population were below the European Union PM_2.5_ limit (25 μg/m^3^) but above the U.S. PM_2.5_ limit (12 μg/m^3^). Furthermore, the effects of PM_2.5_ exposure are potentially transmitted through the *PLCG* and *SOS* signaling cascades. Our molecular epidemiological findings add to recent experimental research suggesting that developmental processes in the placenta and fetal brain are shaped by the same biological signals. However, it is necessary to replicate our results in other study populations. Furthermore, the long-term consequences of these observations remain to be elucidated.

## Supplemental Material

(396 KB) PDF
