# Supplementary material for: In Utero Fine Particle Air Pollution and Placental Expression of Genes in the Brain-Derived Neurotrophic Factor Signaling Pathway: An ENVIRONAGE Birth Cohort Study
Source: Environ Health Perspect. 2015 Mar 27;123(8):834–40. doi: 10.1289/ehp.1408549 (PMC4529006; doi:10.1289/ehp.1408549)
Supplement: (396 KB) PDF [file ehp.1408549.s001.acco.pdf]

Environ Health Perspect
doi: 10.1289/ehp.1408549

Note to Readers: ***EHP*** **strives to ensure that all journal content is accessible to all readers. However, some figures and Supplemental Material published in** ***EHP*** **articles may not conform to 508 standards due to the complexity of the information being presented. If you need assistance accessing journal content, please contact ehp508@niehs.nih.gov. Our staff will work with you to assess and meet your accessibility needs within 3 working days.**

Supplemental Material

In Utero Fine Particle Air Pollution and Placental Expression of Genes in the Brain-Derived Neurotrophic Factor Signaling Pathway: An ENVIR*ON*AGE Birth Cohort Study

Nelly D. Saenen, Michelle Plusquin, Esmée Bijnens, Bram G. Janssen, Wilfried Gyselaers, Bianca Cox, Frans Fierens, Geert Molenberghs, Joris Penders, Karen Vrijens, Patrick De Boever, and Tim S. Nawrot

Table of Contents

Table S1. Primer assays for selected genes and their RefSeq number.

Table S2. Within-placenta and between-placenta variability of the two placental biopsies for each gene.

Table S3. Correlations between genes in the *Bdnf* signaling pathway.

Table S4. Exposure characteristics of NO_2 (n = 90).

Figure S1. Difference in placental gene expression in association with *in utero* exposure to fine particle air pollution ($PM_{2.5}$) during various time windows (single-gene models; n=90). The effect estimates are the percent difference (95% CI) relative to mean gene expression for a 5 µg/m³ increment of $PM_{2.5}$ exposure (µg/m³). Time window specific $PM_{2.5}$ exposures (µg/m³) were calculated by averaging the daily interpolated $PM_{2.5}$ concentrations for various periods during pregnancy: each of the three trimesters. Estimates were adjusted for newborn's gender, maternal

age, maternal education, gestational age, cord blood insulin, placental biopsy site, delivery date, season at birth and NO_2 exposure. * $p < 0.05$

Table S1. Primer assays for selected genes and their RefSeq number.

Abbreviation	Gene	RefSeq number	Primetime® Std qPCR Assay	Efficiency (%)
**Target genes**				
BDNF	Brain-derived neurotrophic factor	NM_001709	Hs.PT.56a.27098180.g	100
TRKB	Neurotrophic tyrosine kinase receptor type 2	NM_001018065	Hs.PT.56a.39058236.g	112
AKT1	V-akt murine thymoma viral oncogene homolog 1	NM_005163	Hs.PT.56a.15697853.g	92
AKT2	V-akt murine thymoma viral oncogene homolog 2	NM_001243027	Hs.PT.56a.143554.g	99
AKT3	V-akt murine thymoma viral oncogene homolog 3	NM_005465	Hs.PT.56a.4178001	92
SOS1	Son of sevenless homolog 1	NM_005633	Hs.PT.56a.20852108	93
SOS2	Son of sevenless homolog 2	NM_006939	Hs.PT.56a.14433335	92
PLCG1	Phospholipase C gamma 1	NM_182811	Hs.PT.56a.2187691	99
PLCG2	Phospholipase C gamma 2	NM_002661	Hs.PT.56a.45287498	99
SYN1	Synapsin 1	NM_006950	Hs.PT.56a.4883078.g	107
**Reference genes**				
GAPDH	Glyceraldehyde-3-phosphate dehydrogenase	NM_001256799	Hs.PT.53a.24391631.gs	100
IPO8	Importine 8	NM_001190995	Hs.PT.56a.40532361	95
UBC	Ubiquitine C	NM_021009	Hs.PT.39a.22214853	95
POLR2A	Polymerase (RNA) II, polypeptide A	NM_000937	Hs.PT.56a.25515089	95

Table S2. Within-placenta and between-placenta variability of the two placental biopsies for each gene.

Gene	Within-placenta variability (%)	Between-placenta variability (%)
BDNF	38.7	61.3
TRKB	42.3	57.7
AKT1	16.1	83.9
AKT2	52.7	47.3
AKT3	39.0	61.0
SOS1	43.7	56.3
SOS2	38.9	61.1
PLCG1	30.2	69.8
PLCG2	53.3	46.7
SYN1	69.3	30.7

Table S3. Correlations between genes in the *Bdnf* signaling pathway.

	BDNF	***TRKB***	***AKT1***	***AKT2***	***AKT3***	***SOS1***	***SOS2***	***PLCG1***	***PLCG2***	***SYN1***
BDNF	1	0.071<br>0.43	-0.48<br><.0001	0.071<br>0.39	-0.23<br>0.004	0.18<br>0.03	0.43<br><.0001	-0.43<br><.0001	0.19<br>0.02	0.19<br>0.03
TRKB		1	-0.07<br>0.42	-0.04<br>0.63	-0.027<br>0.75	-0.0069<br>0.94	0.058<br>0.50	-0.034<br>0.70	-0.021<br>0.82	0.16<br>0.08
AKT1			1	0.33<br><.0001	0.42<br><.0001	-0.10<br>0.21	-0.40<br><.0001	0.74<br><.0001	-0.13<br>0.12	-0.02<br>0.77
AKT2				1	0.65<br><.0001	0.48<br><.0001	0.27<br>0.0004	0.28<br>0.0002	0.43<br><.0001	0.28<br>0.0005
AKT3					1	0.39<br><.0001	0.18<br>0.018	0.36<br><.0001	0.049<br>0.54	0.16<br>0.05
SOS1						1	0.60<br><.0001	0.13<br>0.096	0.54<br><.0001	0.08<br>0.33
SOS2							1	-0.30<br><.0001	0.26<br>0.0012	-0.04<br>0.67
PLCG1								1	0.15<br>0.068	0.07<br>0.39
PLCG2									1	0.31<br>0.0002
SYN1										1

Given is pearson correlation and p-value.

Table S4. Exposure characteristics of NO_2 (n = 90).

	NO_2,ug/m³		
**Time windows**	**Mean ± SD**	**25th percentile**	**75th percentile**
Pre-implantation (1-5d)	21.6 ± 8.9	15.8	25.5
Implantation (6-12d)	21.1 ± 8.1	15.6	25.6
Implantation range[a] (6-21d)	21.0 ± 7.2	15.8	25.0
Post-implantation (22-28d)	19.9 ± 8.2	13.3	24.1
First month (1-30d)	20.4 ± 7.1	15.3	24.4
Trimester 1 (1-13w)	20.3 ± 6.6	15.9	24.3
Trimester 2 (14-26w)	22.3 ± 7.2	16.6	26.4
Trimester 3 (27w-delivery)	23.6 ± 7.3	18.3	28.1

[a]Data available for 79 subjects.

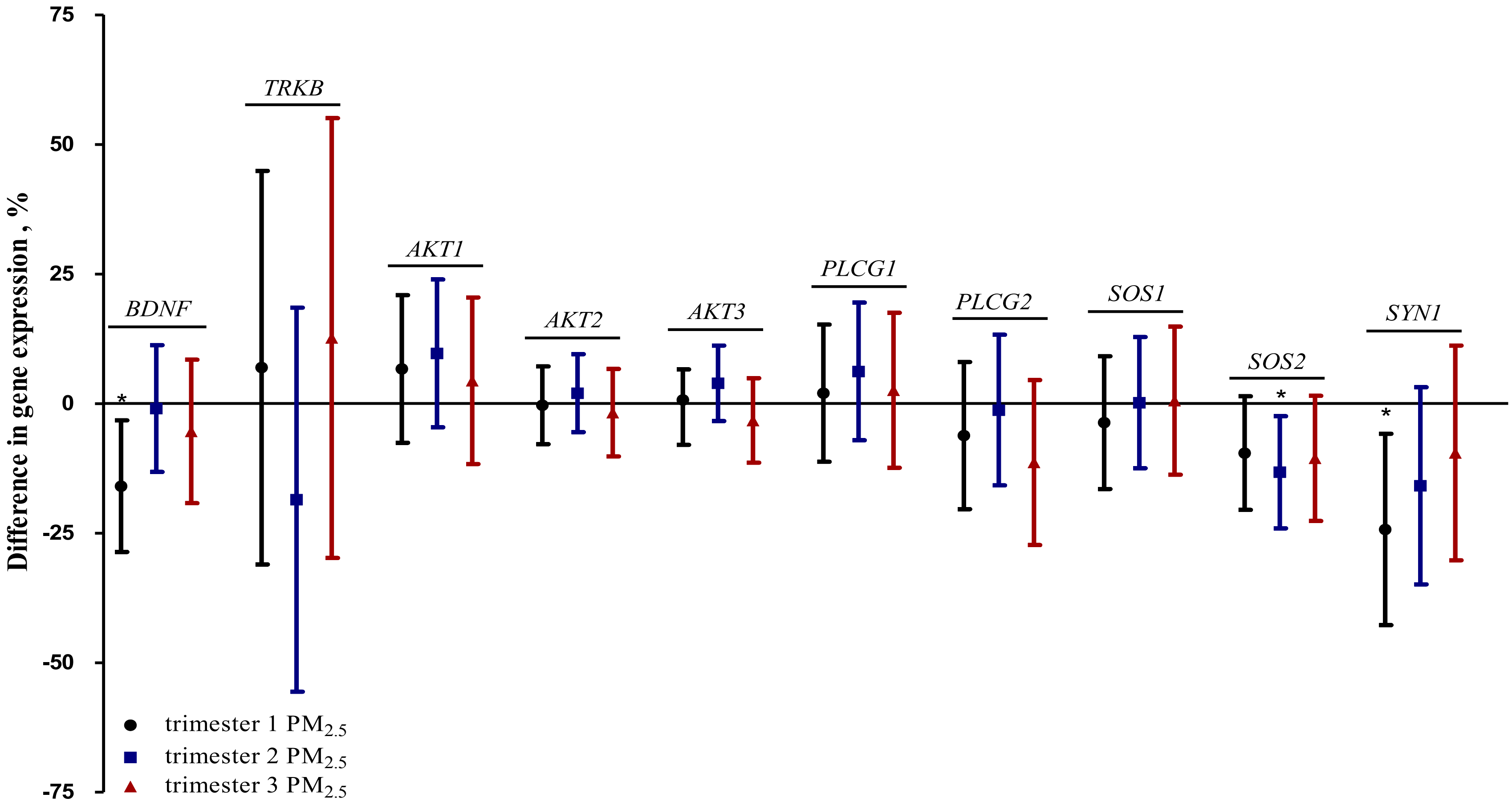

Figure S1. Difference in placental gene expression in association with *in utero* exposure to fine particle air pollution ($PM_{2.5}$) during various time windows (single-gene models; n=90). The effect estimates are the percent difference (95% CI) relative to mean gene expression for a 5 µg/m³ increment of $PM_{2.5}$ exposure (µg/m³). Time window specific $PM_{2.5}$ exposures (µg/m³) were calculated by averaging the daily interpolated $PM_{2.5}$ concentrations for various periods during pregnancy: each of the three trimesters. Estimates were adjusted for newborn's gender, maternal age, maternal education, gestational age, cord blood insulin, placental biopsy site, delivery date, season at birth and NO_2 exposure. * $p < 0.05$
